# Early Loss of Xist RNA Expression and Inactive X Chromosome Associated Chromatin Modification in Developing Primordial Germ Cells

**DOI:** 10.1371/journal.pone.0000860

**Published:** 2007-09-12

**Authors:** Mariana de Napoles, Tatyana Nesterova, Neil Brockdorff

**Affiliations:** Medical Research Council Clinical Sciences Centre, Imperial College Faculty of Medicine, Hammersmith Hospital, London, United Kingdom; Institut Curie, France

## Abstract

**Background:**

The inactive X chromosome characteristic of female somatic lineages is reactivated during development of the female germ cell lineage. In mouse, analysis of protein products of X-linked genes and/or transgenes located on the X chromosome has indicated that reactivation occurs after primordial germ cells reach the genital ridges.

**Principal Findings/Methodology:**

We present evidence that the epigenetic reprogramming of the inactive X-chromosome is initiated earlier than was previously thought, around the time that primordial germ cells (PGCs) migrate through the hindgut. Specifically, we find that Xist RNA expression, the primary signal for establishment of chromosome silencing, is extinguished in migrating PGCs. This is accompanied by displacement of Polycomb-group repressor proteins Eed and Suz(12), and loss of the inactive X associated histone modification, methylation of histone H3 lysine 27.

**Conclusions/Significance:**

We conclude that X reactivation in primordial germ cells occurs progressively, initiated by extinction of Xist RNA around the time that germ cells migrate through the hindgut to the genital ridges. The events that we observe are reminiscent of X reactivation of the paternal X chromosome in inner cell mass cells of mouse pre-implantation embryos and suggest a unified model in which execution of the pluripotency program represses Xist RNA thereby triggering progressive reversal of epigenetic silencing of the X chromosome.

## Introduction

X inactivation, the silencing of one X-chromosome in female mammals, is achieved by the establishment of multiple epigenetic modifications that result in chromatin of the inactive X (Xi) being transcriptionally repressed. Challenging one or several of these modifications can result in sporadic reactivation of single genes, but does not cause reactivation of the whole chromosome [1]–[4]. The stability of X inactivation ensures long-term maintenance of silencing through multiple cell divisions and throughout the lifetime of the animal.

Chromosome wide X-reactivation does occur in certain circumstances, both during normal development, and also in experimental reprogramming of somatic cells. In normal development X reactivation first occurs in pre-implantation blastocyst stage embryos, where paternally imprinted X-inactivation is reversed in inner cell mass (ICM) cells destined to give rise to the embryo proper [5], [6]. X reactivation also occurs during post-implantation stages, specifically in developing primordial germ cells (PGCs) [7]–[9]. In experimental reprogramming X reactivation occurs following stable fusion of XX somatic cells with pluripotent embryonic cells [10], or by transfer of nuclei from XX somatic cells to enucleated unfertilised oocytes [11].

Studies analysing X reactivation in XX PGCs isolated from the genital ridge have shown that at 11.5 days post coitum (dpc), X-linked gene expression can only be detected from one chromosome, while at 13.5 dpc both chromosomes are transcriptionally active [8], [9]. It was concluded that the transcriptional reactivation of Xi in PGCs occurs in the genital ridge at around 13.5 dpc. A more recent report, analysing expression of an X-linked β-galactosidase transgene, indicated that a small percentage of cells reactivate the Xi at 11.5 dpc [12].

Other studies have analysed epigenetic modifications characteristic of the inactive X chromosome. CpG island methylation, a late marker of stable X inactivation [13], [14], was found not to occur in PGCs [15], [16]. Analysis of X inactivation markers in PGCs isolated from genital ridges found that Xist RNA accumulation, the primary mark associated with establishment of X inactivation, is progressively reduced during PGC maturation [17]. Enrichment of the variant histone macroH2A, a late marker of X inactivation [18], was absent at all stages analysed. Here we have extended analysis of X reactivation to migrating PGCs, analysing early X-inactivation markers, recruitment of proteins belonging to Polycomb repressor complex (PRC) 2 [19], methylation of histone H3 lysine 27 (H3K27me3), catalysed by the PRC2 protein Ezh2, and expression/localisation of Xist RNA [20]. Our results demonstrate that PGCs show loss of *Xist* expression and of Xist RNA dependent markers of the inactive X earlier than previously thought, during or immediately prior to migration to the genital ridges.

## Results

### Absence of PRC2 Polycomb-group proteins on Xi in XX primordial germ cells

**Figure 1:**
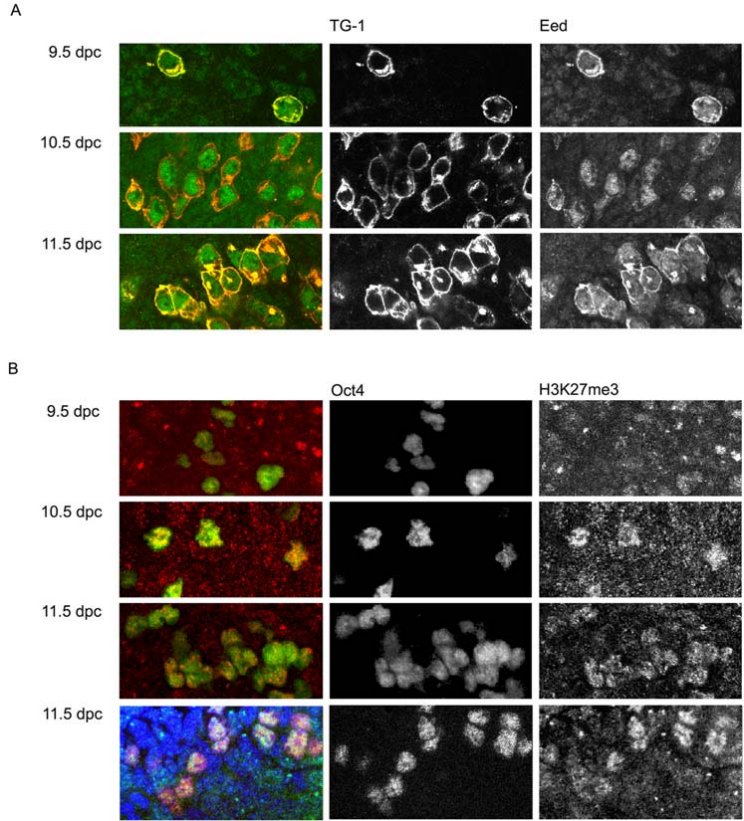
Elevated PRC2 and H3K27me3 levels in developing PGCs. (A) Indirect IF on the hind gut of 9.5 dpc XX embryos and whole genital ridges of 10.5 dpc and 11.5 dpc XX embryos using anti-Eed (in green) and TG-1 (in red/yellow) antibodies. PGCs (TG-1 positive cells) can be easily distinguished from neighbouring cells by their stronger Eed nuclear staining. Both primary antibodies are mouse monoclonals but after sequential detection the signals can be distinguished because TG1 signal is restricted to the cell membrane/cytoplasm and Eed protein is present only in the nucleus. (B) Indirect IF on the hind gut of 9.5 dpc XX embryos and whole genital ridges of 10.5 dpc and 11.5 dpc XX embryos detecting Oct4 (in green) and H3K27me3 (in red). At 9.5 dpc PGCs and neighbouring somatic cells present similar nuclear levels of H3K27me3. At 10.5 dpc and 11.5 dpc, PGCs show much higher levels of this modification than neighbouring somatic cells. The bottom panels show an example where DNA was counterstained with DAPI (blue) to reveal the nuclei of all cells in the section. Images correspond to a single confocal section at 630× original magnification.

We initiated our analysis by assessing the nuclear localisation of the PRC2 protein Eed by indirect immunofluorescence. The presence of single large foci of PRC2 protein or associated H3K27me3 in nuclei of XX cells provides a surrogate marker indicating the presence of Xist RNA [19]. At the onset of random X inactivation in somatic lineages there is a transient high level enrichment of PRC2 proteins on Xi, beginning with initiation of *Xist* expression at 5.5 dpc. By 9.5 dpc PRC2 enrichment is no longer detectable, correlating with a marked reduction in overall levels of PRC2 proteins [19]. Associated H3K27me3, in contrast, persists throughout ontogeny, indicating that low levels of the complex must continue to be recruited to Xi [19]. In a first set of experiments we analysed the whole hindgut region or genital ridges of 9.5 dpc, 10.5 dpc and 11.5 dpc female embryos. For the identification of PGCs we used TG-1 antibodies, which specifically recognise PGCs between 9.5 and 11.5 dpc [20]. We noticed that PGCs could be easily distinguished at all stages based on high levels of Eed in the nucleus relative to neighbouring somatic cells (Figure 1A). The same was observed when the nuclear localisation of Su(z)12, another PRC2 protein, was analysed (data not shown). Despite the high levels of PRC2 proteins in PGCs, we did not observe enrichment on Xi in the majority of cells.

**Figure 2:**
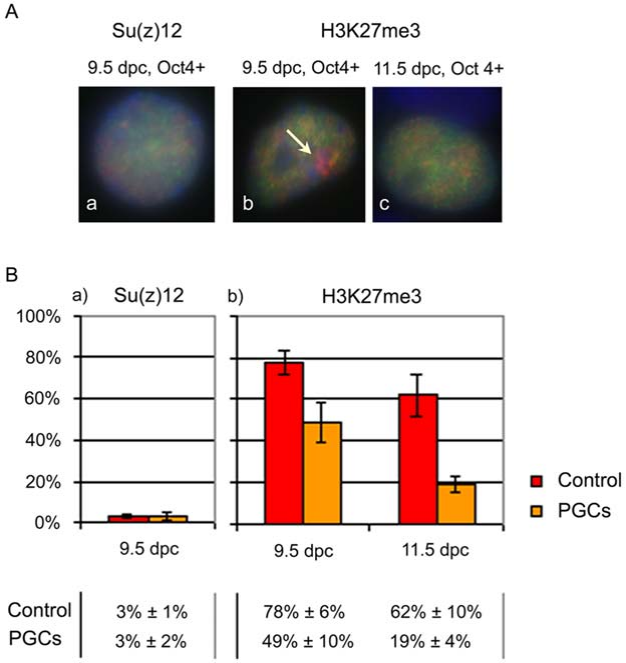
PRC2 and associated H3K27me3 on the inactive X chromosome in developing XX PGCs. (A) a) 9.5 dpc XX PGCs stained for Oct4 in green and Su(z)12 in red. Most PGCs showed diffuse nuclear staining for Su(z)12 and no detectable accumulation corresponding to Xi. b) 9.5 dpc and c) 11.5 dpc XX PGCs stained for Oct4 in green and H3K27me3 in red. At 9.5 dpc approximately 50% of Oct4 positive cells showed H3K27me3 Xi foci (arrow) while at 11.5 dpc most Oct4 positive cells showed only diffuse nuclear staining for H3K27me3 and no detectable accumulation on the Xi. (B) Scoring results for a) Su(z)12, and b) H3K27me3. Plots represent the percentage of Oct4 positive (PGCs) and Oct4 negative (control) cells showing a Su(z)12, or a H3K27me3 Xi signal; error bars represent variation between embryos. Su(z)12 data corresponds to the analysis of 8 embryos/slides; a total of 545 Oct4 positive cells and 1649 Oct4 negative cells were scored. H3K27me3 data corresponds to the analysis of 6 and 7 embryos/slides for 9.5 dpc and 11.5 dpc respectively; a total of 274 (at 9.5 dpc) and 1099 (at 11.5 dpc) Oct4 positive cells were scored, over 1400 cells were scored in controls.

In order to quantify the percentage of 9.5 dpc XX female PGCs showing accumulation of PRC2 on Xi we analysed disaggregated hindgut cells by indirect immunofluorescence using anti-Su(z)12 antibodies. To mark PGCs we used antibodies to Oct4, which under the conditions used gave less non-specific staining of non-PGCs compared to TG-1 antibodies (not shown). Oct4 expression is restricted to PGCs from 9.5 dpc [21]–[23]. The presence of Su(z)12 Xi foci was scored in Oct4 positive (PGC) and Oct4 negative (somatic) cells isolated from 9.5 dpc hindgut regions. The results, illustrated in Figure 2, show that a Su(z)12 Xi signal is present in only 3% of cells in both PGC and control slides. This result was unexpected given that PGCs present relatively high levels of PRC2 proteins, and based on previous studies were expected to have an inactive X chromosome.

### Methylation of H3 lysine 27 on Xi declines between 9.5 and 11.5 dpc in XX PGCs

Our previous studies have demonstrated that low levels of PRC2 enrichment on Xi, undetectable by immunofluorescence, are sufficient to give chromosome wide enrichment of H3K27me3 [19]. We therefore tested if PGCs that lack detectable PRC2 enrichment on Xi also lack H3K27me3 enrichment. Whole-mount staining of hindgut regions from 9.5 dpc embryos revealed that global levels of H3K27me3 are similar in PGCs relative to neighbouring somatic cells (Figure 1B, top panels). In contrast, at 10.5 dpc and 11.5 dpc PGCs showed relatively high nuclear H3K27me3 levels (Figure 1B, lower panels). Elevated H3K27me3 in maturing PGCs has been reported previously [24], and is consistent with the elevated levels of PRC2 components (Figure 1A).

Analysis of the percentage of PGCs presenting H3K27me3 Xi foci was carried out using indirect immunofluorescence on disaggregated hindgut regions or dissected genital ridges. We found that H3K27me3 foci are reduced in PGCs as early as 9.5 dpc, occurring in only 49% of cells compared with 78% in control somatic cells (Figure 2A, B). This percentage was even lower at 11.5 dpc, at which stage only 19% of PGCs showed H3K27me3 accumulation, compared with 62% in controls (Figure 2B). Thus loss of PRC2 enrichment on Xi in 9.5-11.5dpc PGCs is accompanied by progressive loss of associated H3K27me3.

### Extinction of Xist RNA expression in XX PGCs initiates prior to 9.5 dpc

The data obtained for PRC2 proteins and associated H3K27me3 in PGCs is reminiscent of that seen in analysis of inner cell mass (ICM) cells of the developing blastocyst. Here also global PRC2 protein levels are initially elevated with disappearance of Xi localisation coincident with extinction of *Xist* RNA expression. H3K27me3 on Xi persists for a short time and is then also lost [5]. In a separate study it has been shown that both PRC2 recruitment and associated H3K27 methylation depend on ongoing *Xist* expression [25]. With these factors in mind we considered that loss of PRC2 and H3K27me3 enrichment on Xi in migrating PGCs could indicate that *Xist* expression is extinguished at an earlier stage than was previously determined [17]. We therefore decided to reassess *Xist* expression by RNA FISH. For these experiments we used embryos carrying a GFP transgene under the control of the Oct4 promoter. This allowed us to isolate a pure PGC population by FACS sorting, avoiding the need to compromise RNA FISH conditions. Preparations from 9.5 dpc hindgut regions and 11.5 dpc genital ridges from XX embryos were sorted by FACS. The sorted population was cytospun onto slides and immunofluorescence for Oct4 was carried out on a slide from each experiment in order to determine the purity of the population after sorting. We found that between 85% and 96% of cells were positively stained with anti-Oct4 antibodies (data not shown).

**Figure 3:**
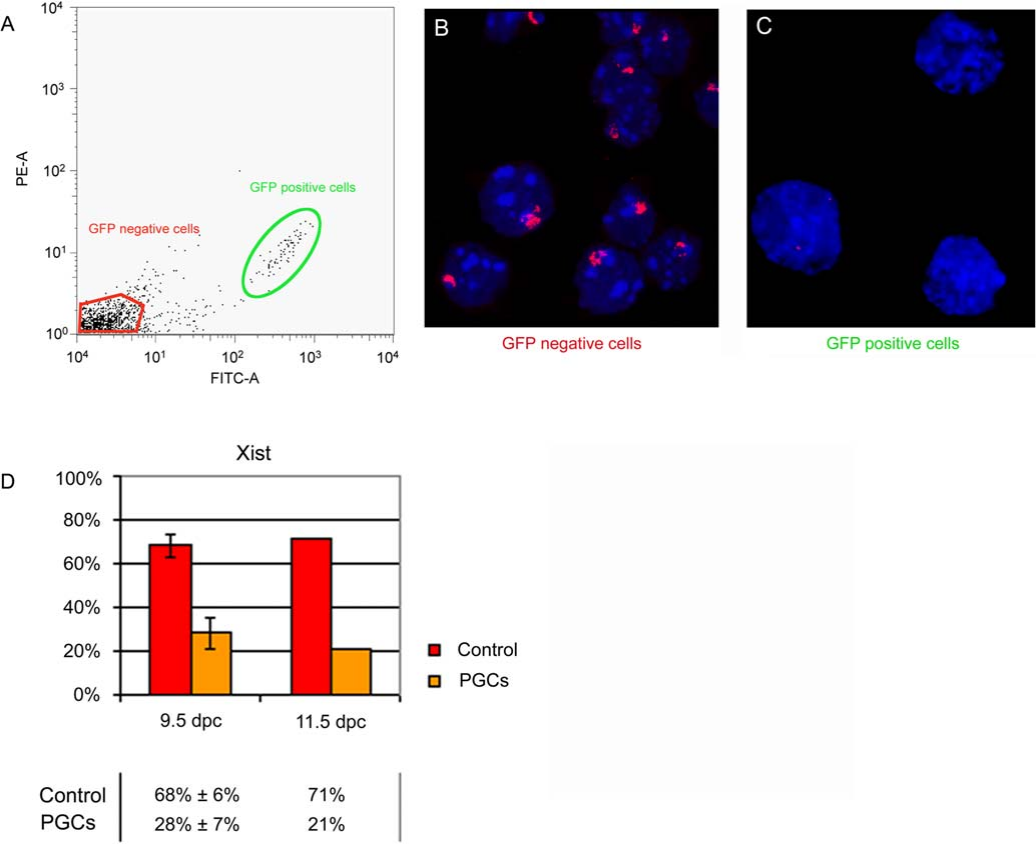
Xist RNA expression in XX PGCs. (A) Example of FACS plot showing GFP-positive and GFP-negative cell populations collected for analysis from dissociated genital ridges of female Oct4-GFP embryos. GFP intensity is shown on the X axis. (B) Example of RNA FISH analysis detecting Xist RNA in the Oct4-GFP negative cells shown in (A). (C) Example of RNA FISH analysis showing Xist RNA domains are absent in the majority of the Oct4-GFP positive PGCs shown in (A). (D) Scoring results showing proportion of cells from XX embryos with Xist RNA domains in Oct4-GFP positive cell (PGCs) compared to unsorted dissociated cell (control) populations. For 9.5 dpc 105 FACS sorted and 405 control cells obtained in two independent experiments were scored. For 11.5 dpc data 140 FACS sorted and 262 control cells were scored.

An example of an experiment with 11.5 dpc genital ridges is shown in Figure 3A. Analysis of Xist RNA by FISH revealed a high proportion of expressing cells in the GFP negative population (Figure 3B). Thus cell sorting per se does not affect detection of Xist RNA. However in GFP positive PGCs we observed markedly reduced numbers of cells showing Xist expression (Figure 3C). We went on to score GFP positive cells from 9.5 and 11.5 dpc XX embryos isolated in independent experiments. For these experiments we scored cells from dissociated hindgut/genital ridge prior to cell sorting as a control. We observed that while approximately 70% of control cells presented an accumulated *Xist* RNA signal, in PGCs only 28% and 21% of cells showed an *Xist* focus at 9.5 dpc and 11.5 dpc, respectively (Figure 3D). Considering somatic contamination in the sorted populations in the range of 4–15% these percentages if anything represent an overestimation.

## Discussion

In this study we demonstrate that loss of *Xist* expression and Xist RNA dependent Xi markers in XX PGCs occurs prior to or during migration of germ cells through the hindgut towards the genital ridge. These early changes, indicative of X chromosome reactivation, preceed detectable expression of Xi genes/transgenes reported previously.

In an earlier study we analysed *Xist* expression in XX PGCs and found that loss of Xist RNA occurs progressively between 10.5 and 13.5 dpc [17]. In light of the results obtained here we have re-assessed the methods used in the earlier study and found that *Xist* signal was often attributable to contaminating somatic cells which, under the conditions used, showed a spurious staining using the TG1 antibody (data not shown). In this study we identified PGCs unequivocally using both TG1 and Oct4 antibodies, and in addition by sorting PGCs expressing the Oct4-GFP transgene. These factors provide strong support for the finding that loss of Xist RNA expression in the majority of PGCs occurs prior to or during germ cell migration at 9.5dpc. However, it is likely that X reactivation in PGCs is not entirely synchronous and that a minority of cells extinguish Xist expression later, following migration to the genital ridges.

Whilst it is clear that extinction of *Xist* expression and loss of Xist RNA dependent chromatin modifications occurs in the majority of PGCs around 9.5dpc, it does not necessarily follow that X reactivation completes at this time. Indeed reactivation of X-linked genes has only been detected in PGCs after they reach the genital ridge at 11.5 dpc [8], [9], [12]. A possible explanation is that reactivation of X-linked genes commences progressively during PGC migration, but that the assays used to date, all of which have analysed protein products, are not sensitive enough to detect this. Developing germ cells in other organisms show general transcriptional repression and there is some evidence that this occurs also in mammals [26], [27]. If so, this could contribute to PGCs showing delayed appearance of gene products from previously inactive X chromosome alleles.

The homeodomain protein Nanog, a marker of pluripotent cells, is expressed in PGCs at 8.0 dpc [28], immediately before the time we surmise *Xist* RNA extinction occurs. This resembles the situation in pre-implantation embryos where Nanog expression first occurs at the morula stage, pre-empting extinction of Xist RNA in ICM cells of the developing blastocyst [5]. Similarly, elevated levels of PRC2 proteins and associated H3K27me3 in PGCs resembles changes in pre-implantation embryos at the time when the paternal X chromosome is reactivated. Based on these parallels we speculate that X-reactivation in PGCs occurs as part of a program to convert the genome to a pluripotent state and that the mechanism for this is similar to that occurring in ICM cells of the developing blastocyst. Thus, similar to the situation in ICM/ES cells, X inactivation in PGCs is likely *Xist* dependent [5], [6], [29]. This contrasts with somatic cells and trophoblast stem (TS) cells where X inactivation is *Xist* independent and essentially irreversible [2], [3], [25], [29], [30].

A number of studies have pointed to the importance of the antisense locus *Tsix* in regulating *Xist* during random and imprinted X inactivation (for review see 31). In our analysis of PGCs we did not observe pinpoint signal indicative of transcription of either Tsix RNA or low levels of Xist RNA from either allele. Based on this we conclude that loss of *Xist* expression in PGCs occurs independently of *Tsix* transcription. Thus, a key issue for future studies is to understand the mechanism by which *Xist* expression is extinguished in pluripotent cells.

## Materials and Methods

### Mouse strains and embryos

Post-implantation mouse embryos were isolated from timed matings of (C57Bl/6 X CBA)F1 animals or (Oct4-GFP X Oct4-GFP) as described previously [19]. Oct4-GFP mice were kindly provided by A. Surani. Embryos were sexed by PCR as described [32]

### Antibodies

For immunofluorescence the following antibodies and dilutions were used: mouse monoclonal anti-Oct4 from Transduction Laboratories (1∶100); TG1, mouse monoclonal, a gift from P. Beverly (undiluted); rabbit polyclonal anti-Su(z)12 from Upstate (1∶200); mouse monoclonal anti-Eed, a gift from A. Otte (1∶100); rabbit polyclonal anti-H3K27me3 from Upstate (1∶200). Secondary antibodies: Alexa 488 and Alexa 568, anti-mouse IgG or anti-rabbit IgG from Molecular Probes.

### Immunofluorescence (IF) and RNA FISH

For the whole mount analysis of 9.5 dpc, 10.5 dpc and 11.5 dpc hind-gut or genital ridges, the region encompassing the hind-gut or genital ridges of each embryo was dissected in 20% FCS medium and washed in PBS. Permeabilisation and blocking were carried out simultaneously by incubating cells for 2 hrs in 0.4 % Triton X-100, 10 mg/ml BSA. Embryo regions were then washed three times in PBS and once in PBS/0.1% Tween-20 for 5 min. Primary antibodies were incubated for 2 hrs in detection buffer (4×SSC, 1 mg/ml BSA, 0.1% Tween-20), except for TG-1, which was used undiluted and incubated for 4 hrs. Primary antibody incubations were followed by three 5 min washes in PBS/0.1% Tween-20. Secondary antibodies were incubated for 1 hr in detection buffer. Embryo regions were subsequently washed three times 5 min in PBS/0.1% Tween-20, rinsed in PBS and mounted on chamber slides in Vectashield antifade containing DAPI. In experiments using both TG1 and anti-Eed antibodies, TG-1 incubation and detection was carried out first. After extensive washing, anti-Oct4 were incubated and detected as described. All steps, except antibody incubations, were carried out in net inserts (Corning incorporated) placed in wells of 24 well plates. Nets, containing the embryonic regions, were transferred between wells and solutions. Antibody incubations were carried out in 50 µl drops of antibody on glass round dishes in a humidified chamber.

For IF on single cells, the region encompassing the hind gut or genital ridges of each embryo was dissected in 20% FCS medium, trypsinised, washed in PBS and resuspended at a concentration of 10^6^ cells/ml in 2% formaldehyde. Cells were fixed for 15 min in a tube and, for each slide, 100 µl of cell suspension was cytospun (cytospin 2, Shandon) in fixing solution for 8 min at 1800 rpm. Slides containing attached cells were washed in PBS, permeabilised for 5 min in 0.4% Triton X-100 in PBS and again washed in PBS. IF was carried out as described [33].

For cell sorting of GFP expressing cells, the hind gut of 9.5 dpc embryos or the genital ridges of 11.5 dpc embryos were dissected in 20% FCS medium and left on ice while embryo heads were processed for sexing by PCR. Embryo regions containing PGCs from female embryos were pooled and then trypsinised in a tube (37°C). Trypsinisation was stopped by the addition of medium, cells were spun down, washed in PBS and resuspended in 500 µl of sort buffer (1×PBS, 1mM EDTA, 25mM Hepes pH 7.0, 1% FCS). Cells were sorted on the basis of their GFP expression level using a FACS VANTAGE SE. Cells in the residual volume of the initial sample were retained and used for controls. The sorted and control cells were washed in PBS and divided in two. Half were used in IF using anti-Oct4 antibodies, and the other half were analysed by RNA FISH. For IF cells were resuspended in 100 µl of 2% formaldehyde/PBS. Fixed for 15 min in a tube, cytospun and processed as described above. For RNA FISH, cells were washed in PBS in an eppendorf tube, spun down, resuspended in 100 µl of ice-cold fixative solution (4% formaldehyde, 5% acetic acid, 0.9% NaCl), and incubated on ice for 25 min. The cell suspension was then cytospun for 8 min at 1800 rpm. Slides with cells were rinsed in PBS and stored in 70% EtOH at 4° C. The *Xist* RNA FISH protocol has been described previously [34], [35]. *Xist* probe preparation was carried out as described [19]. Images were acquired on a Leica DMRB microscope equipped with a CCD camera or on a Leica SP1 confocal microscope.
